# Leather Shaving Waste Extract as an Electrochemical Modifier at a Pencil Graphite Electrode for Paracetamol Determination in Pharmaceuticals

**DOI:** 10.1021/acsomega.4c08502

**Published:** 2025-04-29

**Authors:** Wael Bosnali, Şeyma Korkmaz, Ayşen Demir Mülazımoğlu, İbrahim Ender Mülazımoğlu

**Affiliations:** 1 Institute of Science, Chemistry Department, Necmettin Erbakan University, Konya 42090, Türkiye; 2 Ahmet Keleşoğlu Education Faculty, Chemistry Department, Necmettin Erbakan University, Konya 42090, Türkiye

## Abstract

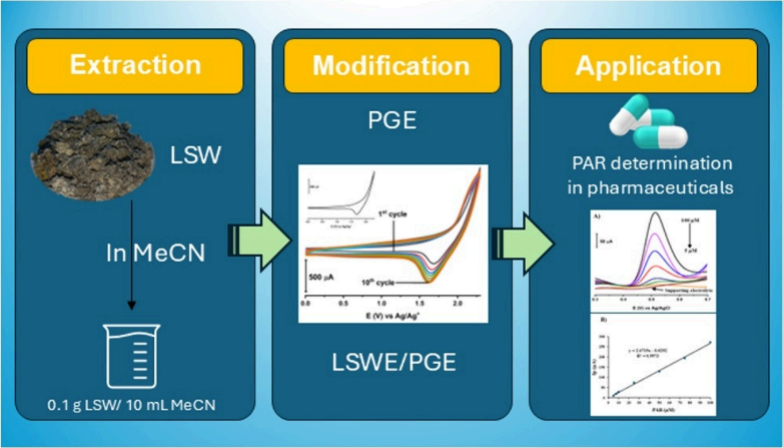

A simple, facile, and sensitive method based on leather shaving waste extract (LSWE) modified pencil graphite electrode (PGE) was developed to determine paracetamol (PAR) by employing the square wave adsorptive stripping voltammetry (SW-AdSV) technique. Leather shaving waste (LSW) was characterized by energy-dispersive X-ray spectroscopy and by investigating its morphology by taking scanning electron microscopy (SEM) images. The extraction process was conducted on an LSW by utilizing acetonitrile. Furthermore, the extraction ratio of LSW to acetonitrile was optimized and found to be 0.1 g LSW/10 mL acetonitrile at room temperature for an extraction period of 12 h. Modification of PGE by 0.1 g of LSWE (0.1LSWE/PGE) was done by performing cyclic voltammetry (CV) at the potential range 0–(+2.3) V for 10 cycles, followed by a characterization process of 0.1LSWE/PGE by employing CV, electrochemical impedance spectroscopy, and SEM techniques. PAR determination parameters at 0.1LSWE/PGE were optimized and found to be an accumulation time of 35 s in Britton Robinson buffer solution at pH 1.8. A linear relationship (*r*^2^ = 0.997) was observed between peak current and PAR concentration within the range 5–100 μM, with a sensitivity of 196.46 μA μM^–1^ cm^–2^. The limit of detection and limit of quantification were found to be 1.6 and 4.51 μM, respectively. Neglected interferant influence on the determination of PAR at 0.1LSWE/PGE was observed in the presence of dopamine, uric acid, caffeine, ascorbic acid, Na^+^, K^+^, Mg^2+^, Ca^2+^, NO_3_^–^, and Cl^–^ ions. In order to evaluate 0.1LSWE/PGE in the determination of PAR in real pharmaceutical samples, different common PAR-containing pharmaceuticals in Türkiye were analyzed, achieving a recovery range of 99.76–102.87%.

## 1 Introduction

Leather production is considered one of the oldest industries ever, which contributes to the release of environmentally hazardous wastes in either solid or liquid form.^1−3^ However, the leather industry sector continues to experience steady growth due to the high consumption of leather products, particularly shoes, garments, and belts.^1^ Raw leather hides are produced as a byproduct of the meat industry, followed by slaughtering of various animals such as cows, sheep, and goats.^1,2^ Leather hides are subjected to a tanning process to preserve them and acquire the desired quality.^4^ The tanning process bonds the carboxylic groups that exist in the collagen structure by utilizing a variety of tannery chemicals like metal ions, aldehydes, and plant extracts.^5^ Chromium ion is the most used tanning material owing to the outstanding characteristics it provides to the final product.^4^ Chromium-tanned leather waste has significant harmful environmental impacts upon disposal in landfills, which makes finding effective recycling methods for that waste an urgent need to prevent its hazardous influence on soil and underground water.^6−8^ Plenty of methods are mentioned in the literature focusing on the recycling of leather waste and suggesting diverse solutions to address this environmental issue. Preparation of packaging materials,^9,10^ biogas generation,^11−13^ preparation of fertilizers,^14−16^ organic material adsorption,^17−19^ and metal removal^20−22^ are examples of some routes that have been developed to recycle and valorize leather wastes. Regarding leather waste application in electrochemistry, activated carbon produced from leather waste showed a supercapacitance property in some studies.^23,24^ Moreover, leather waste was used to prepare a chemical ion detection sensor.^25^ Leather waste has not been used in electroanalytical chemistry to date, and that is the novelty of this study.

**Figure 1:**
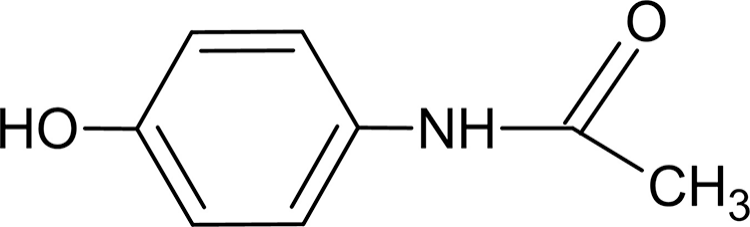
PAR chemical structure.

Paracetamol (PAR) is an analgesic and antipyretic drug commonly used as a pain reliever, particularly prescribed for toothaches, headaches, migraines, muscle pain, and postoperative pain. PAR has an alternative name, acetaminophen (*N*-acetyl-*p*-aminophenol), which is considered a generic name in some countries. PAR contains three main functional groups responsible for its chemical properties and biological effects, as mentioned in Figure 1). The hydroxyl group can form hydrogen bonds with other molecules, which leads to an increase in solubility and reactivity. The amide group is responsible for PAR’s painkilling and fever treatment properties, and a benzene ring provides stability for the PAR molecule. The arrangement of these three functional groups gives PAR its distinctive therapeutic effect.^26−28^ Consumption of PAR may induce several side effects in certain individuals, such as nausea, vomiting, loss of appetite, and abdominal pain. Side effects of consuming PAR-containing pharmaceuticals have been associated with more severe complications, including allergic reactions, liver damage, and kidney damage.^29−31^ Selective quantification of PAR in various pharmaceutical formulations is essential due to the heterogeneity of matrices containing PAR. Consequently, PAR was determined by different analytical techniques such as high-performance liquid chromatography (HPLC),^32^ liquid chromatography–mass spectrometry (LC-MS),^33^ gas chromatography–mass spectrometry (GC-MS),^34,35^ spectrophotometry,^36^ colorimetry,^37^ and chemiluminescence.^38^ Those methods are time-consuming and high-cost. Electrochemical methods are also investigated in the determination of PAR through performing different electrochemical techniques such as differential pulse voltammetry (DPV),^39,40^ square wave voltammetry (SWV),^41^ square wave anodic stripping voltammetry (SWASV),^42^ and linear sweep voltammetry (LSV).^43^ Electrochemical methods offer superior sensitivity, lower cost, and reduced analysis time compared to conventional methods. A range of electrode materials has been investigated recently for PAR detection, demonstrating diverse modification strategies. Examples include: activated 3D-printed electrode (E-3D),^44^ furoic acid-doped-overoxidized poly(3,4-ethylenedioxythiophene)-modified pencil graphite electrode (furoic acid-doped-oo-PEDOT/PGE),^45^ cork-modified carbon paste electrode,^46^ nanodiamond modified film glassy carbon electrode (ND/GCE),^47^ nanocomposite of rGO and TiO_2_ nanoparticles (TiO_2_/rGO) electrode,^48^ and screen-printed electrode decorated with low content Pt–Ni microstructures (Pt–Ni/SPE).^49^

Herein, this study presents the pioneering application of leather shaving waste (LSW) in the field of electroanalytical chemistry. LSW was subjected to an extraction process with acetonitrile, followed by modification of PGE by the resulting leather shaving waste extract (LSWE). The electroanalytical performance of the LSWE modified PGE for the determination of PAR was subsequently studied. The modified electrode showed exceptional sensitivity and a low detection limit, highlighting its promising potential for PAR detection. This innovative electrode leverages readily available materials by employing common pencil leads as PGE and a waste extract, to modify the electrode and increase its electrochemical properties. Consequently, this electrode is considered cost-effective and obtainable worldwide compared to conventional electrodes that require expensive pure chemicals.^50−53^ Also, the construction of this electrode promotes waste consumption and recycling of LSW, opening new opportunities for LSW applications in electroanalytical chemistry.

## 2 Materials and Methods

### 2.1 Materials

The following materials were used in conducting this study: paracetamol (Sigma-Aldrich, 99%), ferrocene (Aldrich, 98%), acetonitrile (BDH, 99.9%), tetrabutylammonium tetrafluoroborate (NBu_4_BF_4_) (Fluka, 98%), a mixture of ferricyanide/ferrocyanide (Fe(CN)_6_^3–/4–^) (PS Park, 98%), potassium chloride (Ridel-de Haen, 99.5%) boric acid (Merck, 100%), acetic acid (Merck, 100%), phosphoric acid (Merck, 85%), ascorbic acid (Scharlau, 99.7%), uric acid (Alfa Aesar, 99%), dopamine (Sigma, 99%), caffeine (Aldrich, 99%), sodium chloride (PS Park, 99.5%), calcium nitrate (PS Park, 98%), potassium chloride (Ridel-de Haen, 99.5%), and magnesium nitrate (Sigma, 99%). All chemicals used in this research were of analytical-grade purity.

### 2.2 Preparation of Solutions

Pure acetonitrile was used in the extraction process of LSW and utilized as a solvent in a nonaqueous medium. The BR buffer solution was prepared by mixing certain amounts of boric acid, acetic acid, and phosphoric acid as mentioned in ref (54). 100 mM potassium chloride was used as a supporting electrolyte in aqueous medium in the characterization process. 100 mM tetrabutylammonium tetrafluoroborate (NBu_4_BF_4_) was used as supporting electrolyte in nonaqueous medium. One mM mixture of ferricyanide/ferrocyanide (Fe(CN)_6_^3–/4–^) solution was used for electrochemical impedance measurements. One mM Ferrocene was used in the characterization of the electrode. All aqueous solutions were prepared using ultrapure water with a resistivity of 18.2 MΩcm (mp MINI pure DEST up).

### 2.3 Instruments

Potentiostat/galvanostat/ZRA (Gamry, USA, model reference 600+) was used in all voltammetric measurements. A three-electrode cell system equipped with Ag/AgCl/3 M KCl (BASi, USA, model MF-2056) and Ag/Ag^+^/10 mM AgNO_3_ (BASi, USA, model MF-2062) was used as reference electrodes in aqueous and nonaqueous media, respectively. In addition, a platinum wire (BASi, USA, model MW-1032) was used as a counter electrode. A cell stand (BASi, USA, model C-3) was used as a Faraday cage integrated with a magnetic stirrer for accumulation voltammetric measurements. 0.7 mm common pencil leads were utilized as a working electrode in bare and modified forms. SEM (ZEISS, Germany, model Gemini 500) and FT-IR (Thermo Scientific–Nicolet, USA model iS20) were utilized to characterize LSW and LSWE.

### 2.4 LSWE Preparation

LSW was collected from a shoe factory situated in Çorum, Türkiye. An amount of the collected sample was cleaned in ultrapure water by magnetic stirring to eliminate all water-soluble impurities and salts, followed by filtration and drying at 50 °C until reaching a constant weight. Different amounts of dried, clean LSWs (0.1, 0.2, 0.3, 0.4, and 0.5 g) were immersed in 10 mL of pure acetonitrile for 12 h at room temperature in a closed container. After that, the LSWE was decanted into another container. The prepared extract solutions were subjected to a characterization process to identify the optimal ratio of LSW to the solvent acetonitrile during the extraction process.

### 2.5 Real Sample Preparation

A tablet of PAR-containing pharmaceuticals was ground by using a mortar and pestle into a fine powder. Subsequently, a certain mass of the ground tablet was dissolved in BR buffer solution at pH 1.8 in a volumetric flask. The final concentration of PAR in the prepared samples was 50 μM, depending on the claimed concentration by the manufacturer.

## 3 Results and Discussion

### 3.1 Characterization of LSW and LSWE

**Figure 2:**
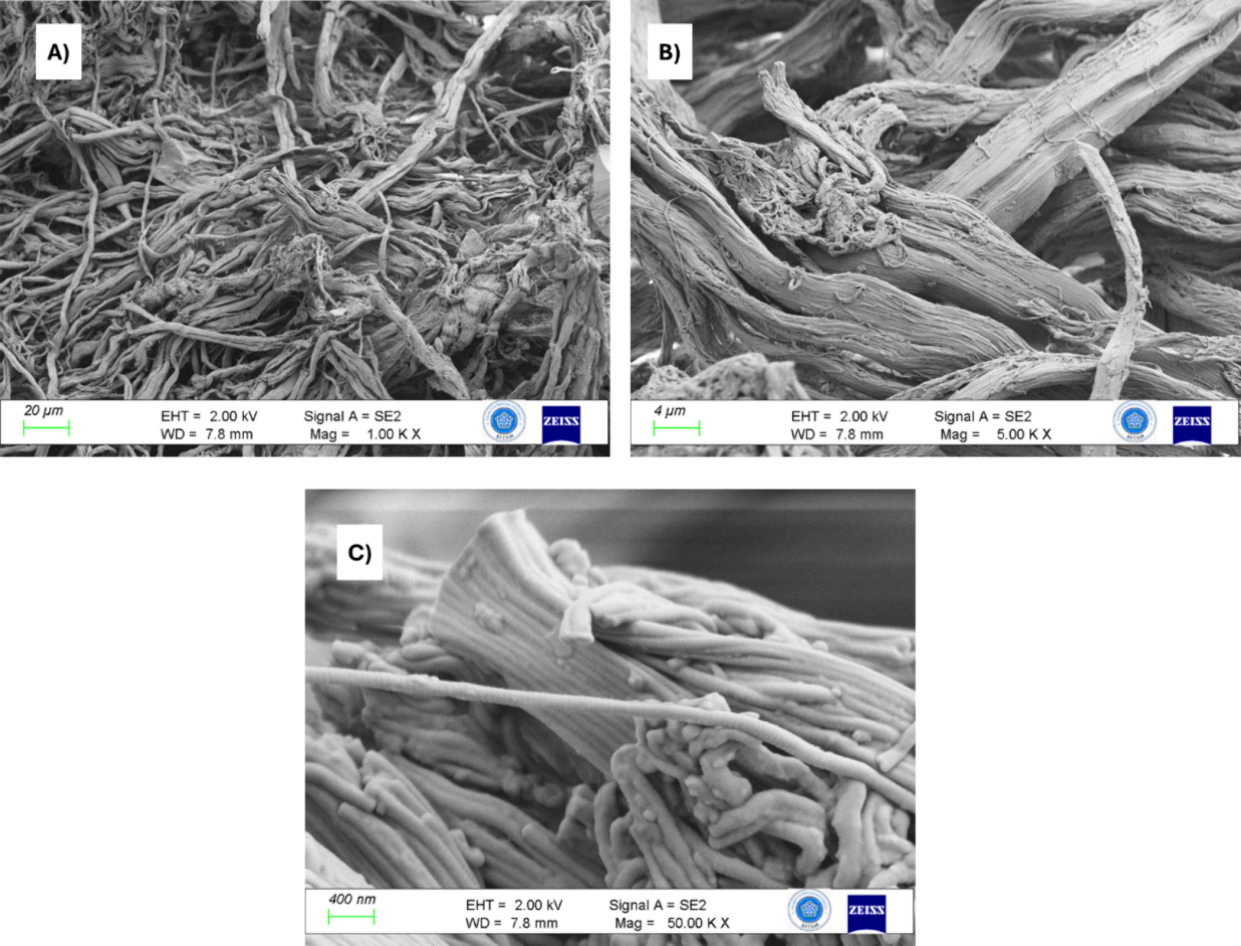
SEM images for LSW at different magnifications. (A) 1000×, (B) 5000×, and (C) 50,000×.

LSW was characterized by SEM and EDX techniques, and LSWE was characterized by IR spectroscopy. Figure 2 illustrates SEM images of LSW at different magnifications. These images reveal the homogeneity of the leather fibers within the sample.

**Figure 3:**
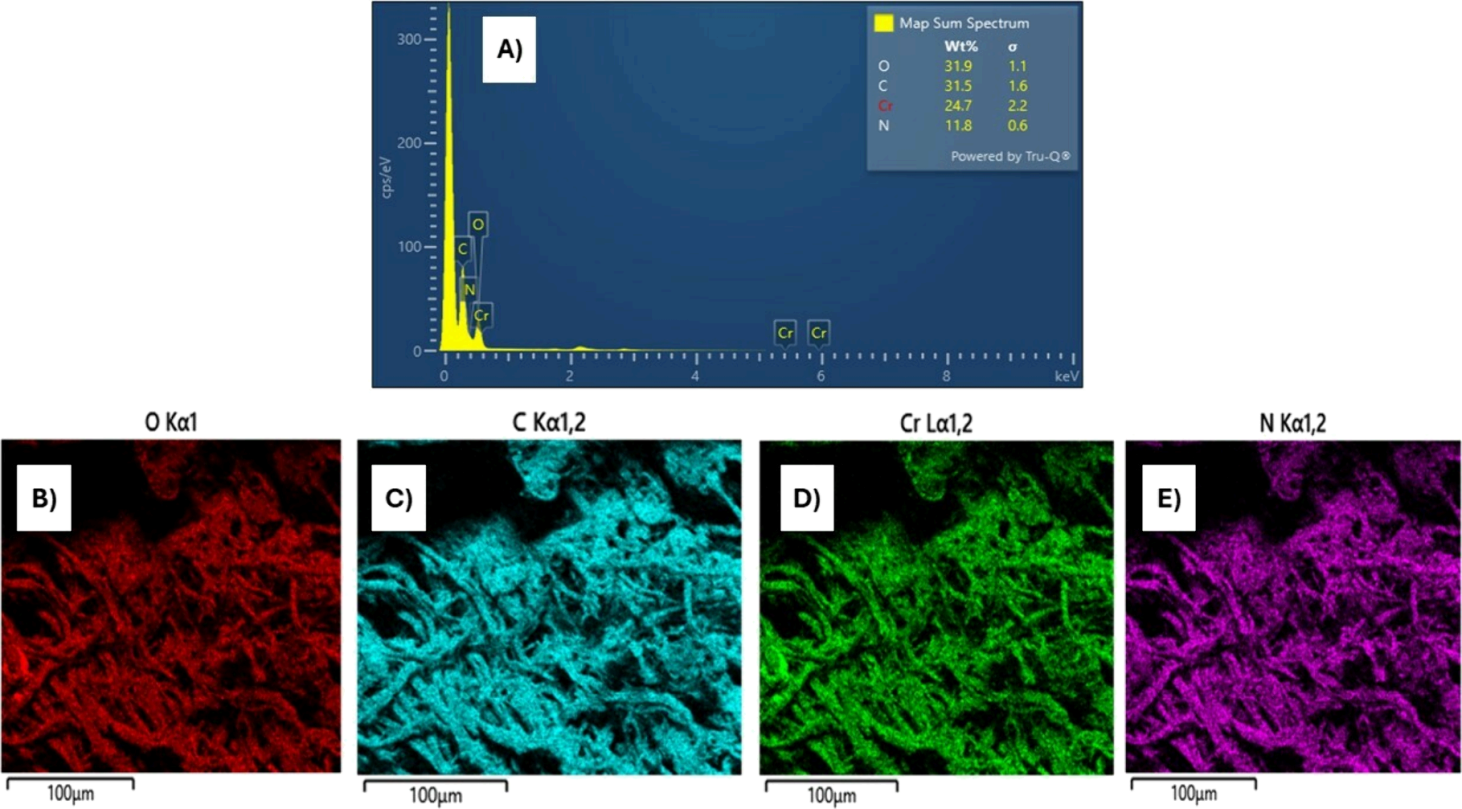
(A) EDX spectrum for LSW. (B–E) Corresponding element mapping images of O, C, Cr, and N for LSW, respectively.

The EDX spectrum for LSW is shown in Figure 3A, revealing its elemental analysis. Oxygen, carbon, chromium, and nitrogen were detected in the LSW sample at the following weight percentages: 31.9, 31.5, 24.7, and 11.8%, respectively. The elevated chromium content in the LSW sample suggests that LSW underwent a tanning process with a chromium ion. Figure 3B–E demonstrates the element mapping images of O, C, Cr, and N, respectively. These images confirm the uniform distribution of these elements within the LSW sample.

**Figure 4:**
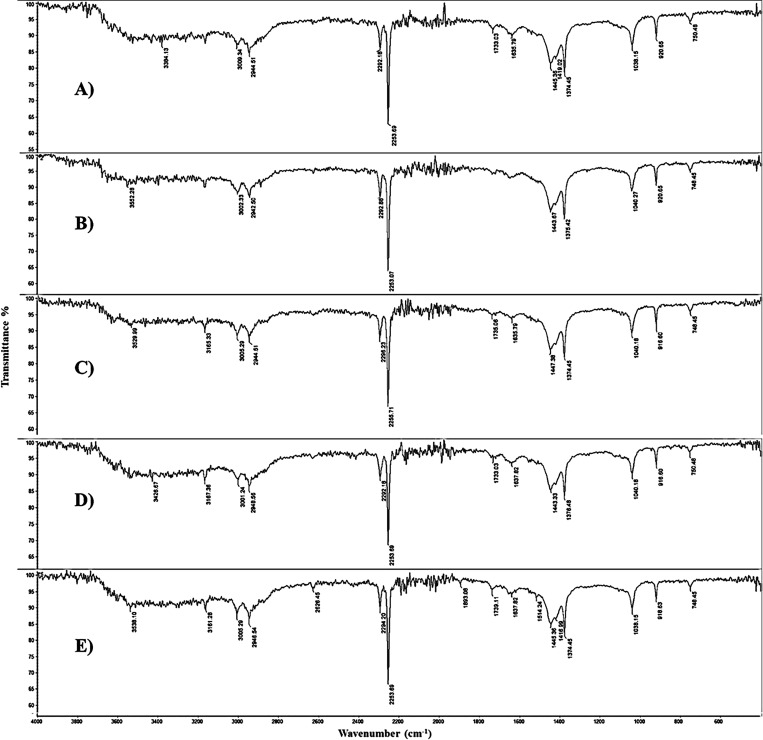
IR spectra for (A) 0.1LSWE, (B) 0.2LSWE, (C) 0.3LSWE, (D) 0.4LSWE, and (E) 0.5LSWE.

IR spectra of LSWE at various extraction ratios of 0.1, 0.2, 0.3, 0.4, and 0.5 (g) LSW/10 mL acetonitrile, which correspond to 0.1LSWE, 0.2LSWE, 0.3LSWE, 0.4LSWE, and 0.5LSWE, respectively, are demonstrated in Figure 4. The IR spectra exhibited identical bands across all extraction ratios, in either position or intensity. Characteristic IR bands of LSWE in all extraction ratios were small single peaks at 3530 cm^–1^ and 3160 ν(−NH_2_), medium single peak at 2250 cm^–1^ ν(−CN), weak double peaks at 1445 and 1374 cm^–1^ ν(C–H), weak single peak at 1030 cm^–1^ ν(C–O), and weak single peak at 1030 cm^–1^ ν(C–O–C). The detection of the NH_2_ functional group in the LSWE, as evidenced by EDX analysis, suggests the electrochemical activity of LSWE. The presence of the (−NH_2_) group in the LSWE is likely to contribute to its electrochemical activity. (−NH_2_) groups can exhibit redox behavior and may facilitate electron transfer processes. Furthermore, these functional groups can potentially interact with the PGE surface, forming bonds that enhance the electrode’s surface area. This increased surface area can lead to improved electron transfer kinetics and enhanced electrochemical performance. 0.1LSWE was selected for further investigations since it provides equivalent efficacy while exhibiting the lowest LSW to acetonitrile ratio in comparison to other extraction ratios.

### 3.2 Modification of PGE by 0.1LSWE

**Figure 5:**
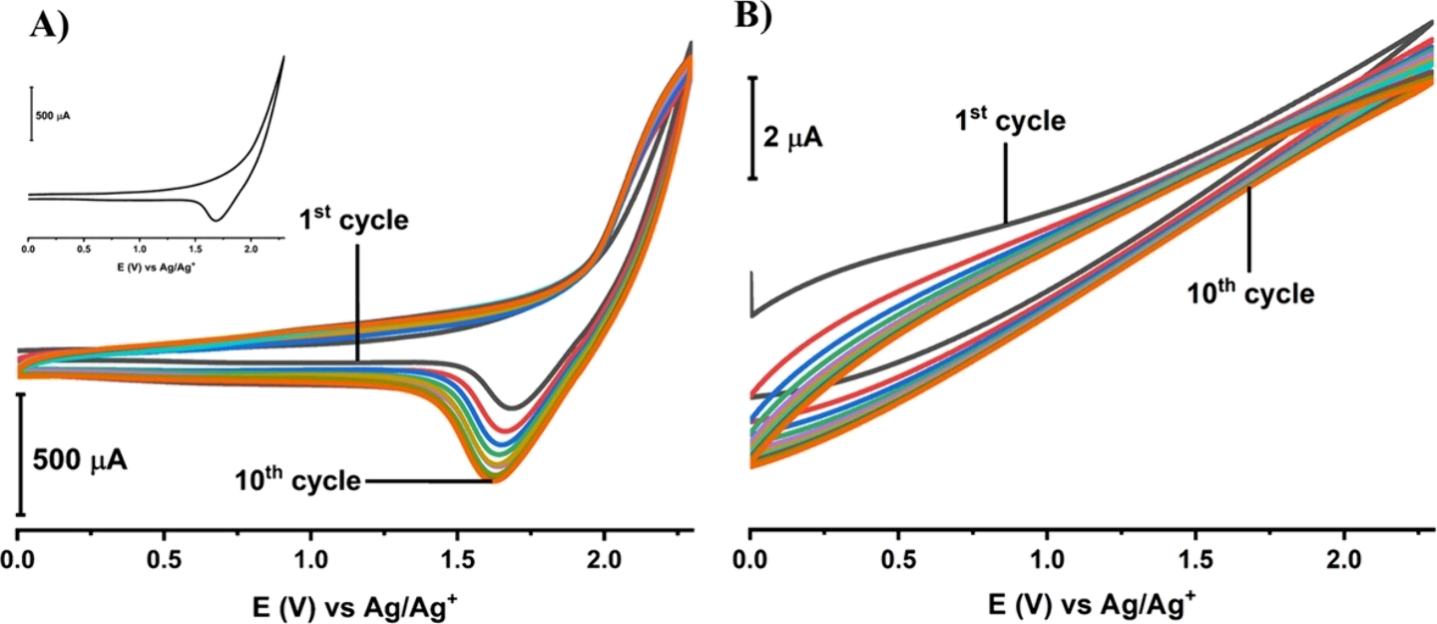
(A) Cyclic voltammogram of 0.1LSWE in the presence of 100 mM NBu_4_BF_4_ versus Ag/Ag^+^/(10 mM AgNO_3_) on PGE at the potential range from 0 to +2.3 V for 10 cycles. Inset: voltammogram of the first cycle. (B) Cyclic voltammogram of acetonitrile versus Ag/Ag^+^/(10 mM AgNO_3_) on PGE at the potential range from 0 to +2.3 V for 10 cycles.

The modification of PGE by 0.1LSWE in the presence of 100 mM NBu_4_BF_4_ as a supporting electrolyte was conducted by performing the CV technique at a potential range from 0 to +2.3 V for 10 cycles. A distinct reduction peak was observed at approximately 1.6 V in the modification voltammogram (Figure 5A). The reduction peak response exhibits a gradual rise until the eighth cycle, followed by the same response at the next cycles, which indicates sufficient 10 cycles of modification to modify the PGE by 0.1LSWE and ensures the complete filling of existing pinholes on the PGE for a homogeneous surface morphology. This reduction peak could be associated with the presence of a reducible functional group that exists in the LSWE. Cyclic voltammetry at PGE in acetonitrile showed no redox peaks (Figure 5B). Consequently, the peaks in Figure 5A belong to 0.1LSWE.

### 3.3 Characterization of LSWE/PGEs

**Figure 6:**
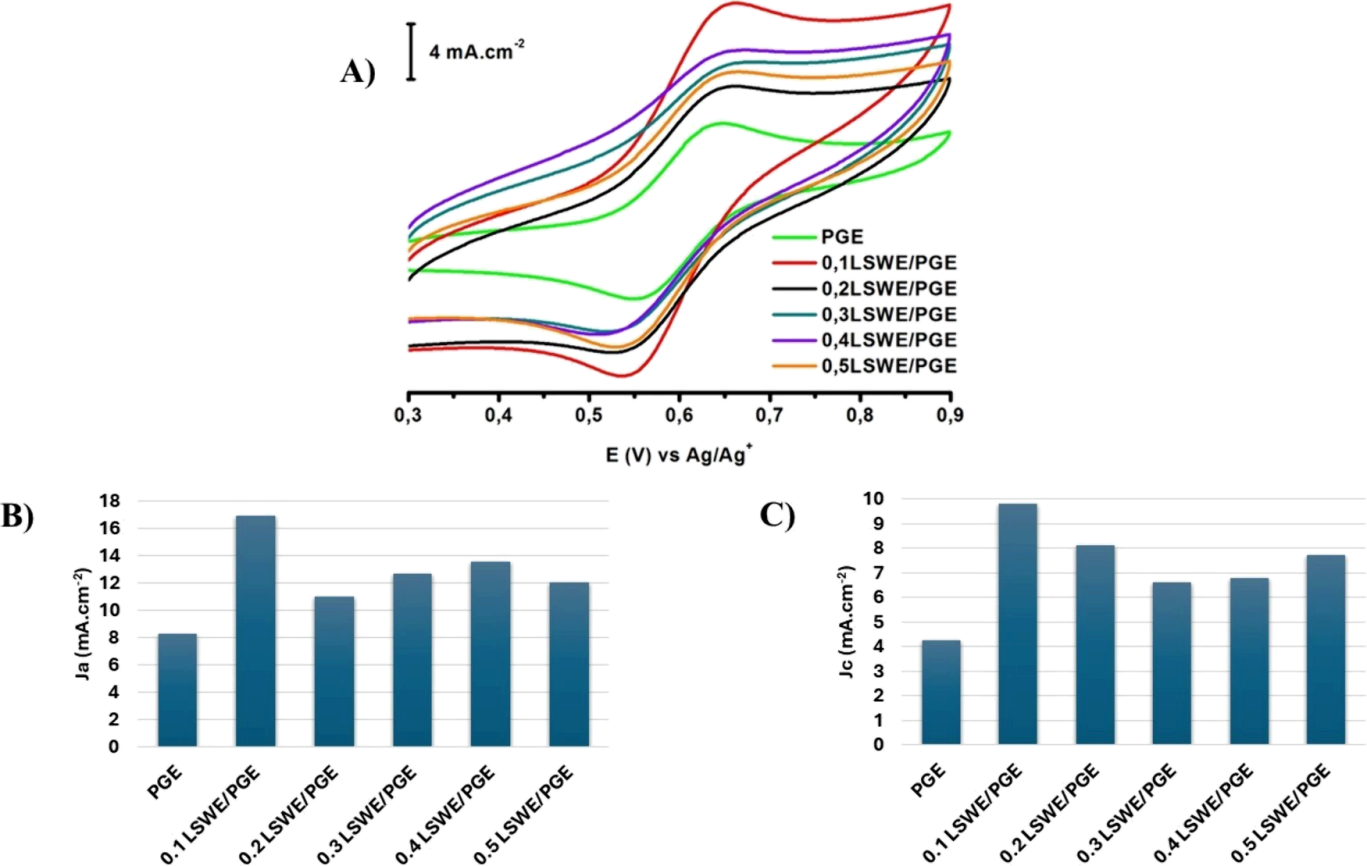
(A) Cyclic voltammogram of 1 mM ferrocene in acetonitrile in the presence of 100 mM NBu_4_BF_4_ at 0.1, 0.2, 0.3, 0.4, and 0.5LSWE/PGEs and PGE. Bar charts of relevant current densities of 0.1, 0.2, 0.3, 0.4, and 0.5LSWE/PGEs and PGE for (B) anodic and (C) cathodic peaks.

EIS, CV, and SEM techniques were performed to characterize the fabricated electrodes, followed by the selection of the best electrode to be used for further investigations. Five electrodes were fabricated by modification of PGE with different LSWE ratios of 0.1, 0.2, 0.3, 0.4, and 0.5 g of LSW/10 mL of acetonitrile. Figure 6A shows CV voltammograms of 1 mM ferrocene in the presence of 100 mM NBu_4_BF_4_ at fabricated electrodes in comparison to PGE. Furthermore, Figure 6B,C illustrate relevant bar charts of anodic and cathodic current densities (μA cm^–2^), respectively. Electrochemical responses of the modified electrodes exhibited sharp enhancement in both cathodic and anodic peaks when 0.1LSWE/PGE was used, and that might be attributed to the formation of a more precise modification layer. However, further modification with higher LSWE ratios (0.2–0.5LSWE) resulted in fluctuating anodic and cathodic responses. All fabricated electrodes showed a higher response to ferrocene than PGE in anodic and cathodic peaks, which indicates that the modification of PGE by LSWE increases its electrochemical activity in nonaqueous medium. 0.1LSWE/PGE showed the best electrochemical activity among other electrodes.

**Figure 7:**
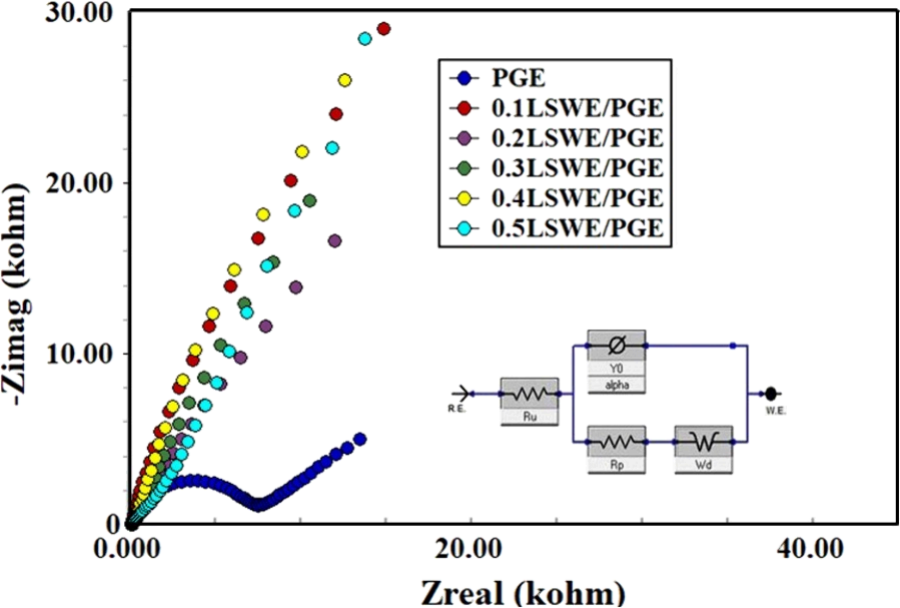
Nyquist type impedance curves of 0.1, 0.2, 0.3, 0.4, 0.5LSWE/PGEs and PGE in a mixture of 1 mM ferricyanide/ferrocyanide Fe(CN)_6_^3–/4–^ solutions in the ratio (1:1) prepared in 100 mM KCl at frequency range 100.000–0.05 Hz and 10 mV wave amplitude.

Electrochemical impedance measurements for 0.1, 0.2, 0.3, 0.4, and 0.5LSWE/PGEs and PGE were conducted in a redox probe solution of 1 mM Fe(CN)_6_^3–/4–^ prepared in 100 mM KCl at a frequency range of 100.000–0.05 Hz and 10 mV wave amplitude. Figure 7 illustrates that 0.1, 0.2, 0.3, 0.4, and 0.5LSWE/PGEs have lower charge transfer resistance than PGE due to the vanishing of the semicircle compared to the presence of PGE’s semicircle in EIS curves. 0.1LSWE/PGE demonstrates a higher curve slope than 0.2, 0.3, 0.4, and 0.5LSWE/PGEs. Consequently, 0.1LSWE/PE possesses superior electroactivity than 0.2, 0.3, 0.4, and 0.5LSWE/PGEs and PGE. Resistance of charge transfer (*R*_CT_) and capacitance (*C*) were determined from EIS curves using Gamry Echem Analyst software and are presented in Table 1. The results in Table 1 suggest that the modification with LSWE significantly impacts the electrochemical properties of the PGE, likely due to a combination of enhanced conductivity, increased surface area, and altered surface properties.

**Table 1:** Comparison in *R*_CT_ (ohm) and *C* (μF) among PGE and 0.1, 0.2, 0.3, 0.4, and 0.5LSWE/PGEs

electrode	*R*_CT_ (ohm)	*C* (μF)
PGE	140.8	1.88
0.1LSWE/PGE	64.08	296.9
0.2LSWE/PGE	58.78	394.9
0.3LSWE/PGE	79.07	415.5
0.4LSWE/PGE	76.38	336
0.5LSWE/PGE	105.7	238.1

**Figure 8:**
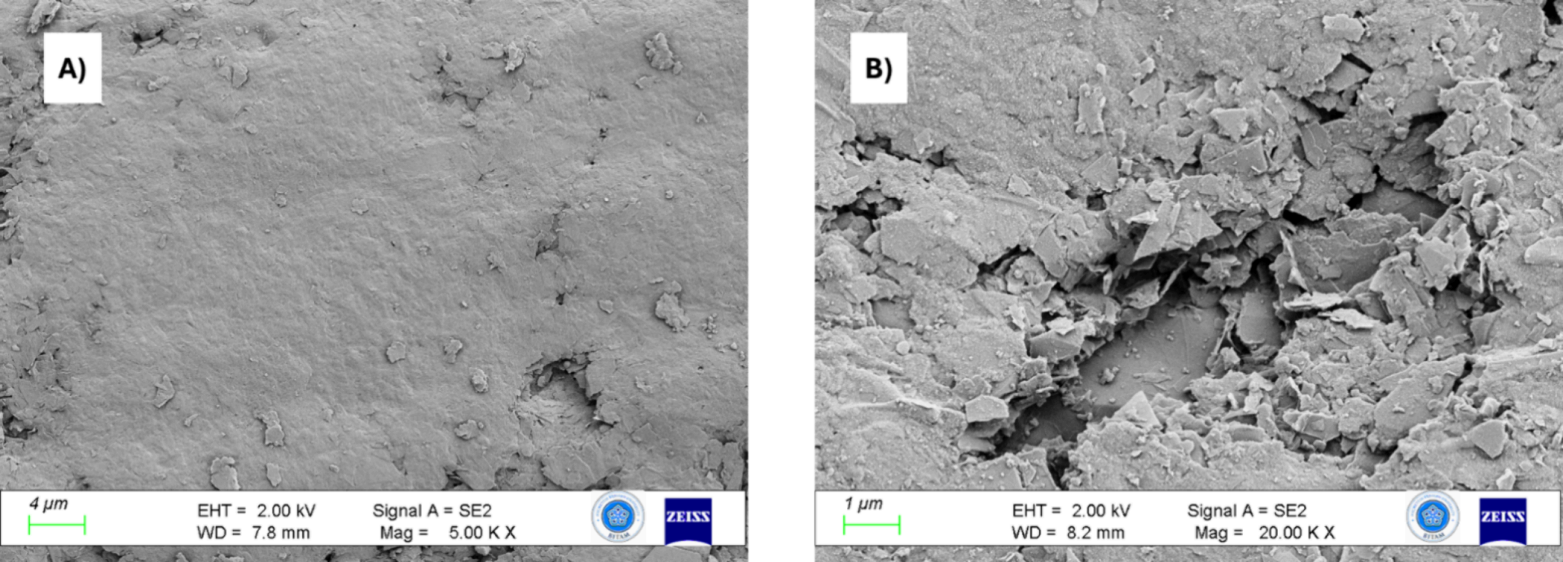
SEM images for (A) PGE and (B) 0.1LSWE/PGE.

SEM images for PGE and 0.1LSWE/PGE in Figure 8 indicated the morphological alterations on the PGE surface upon modification by 0.1LSWE.

### 3.4 Application of 0.1LSWE/PGE in the Determination of PAR

**Figure 9:**
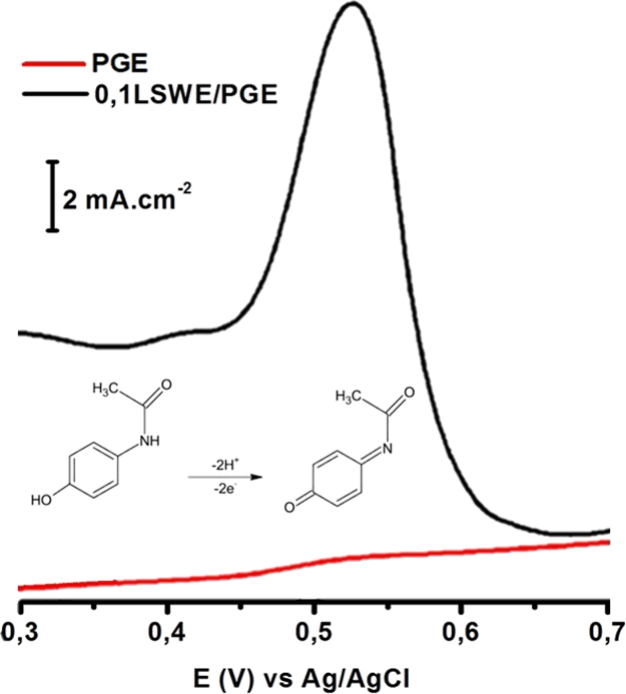
SW-AdS voltammograms of 100 μM PAR in BR buffer solution at pH 1.8 and 35 s accumulation time at 0.1LSWE/PGE and PGE.

The electrochemical sensing performance of 0.1LSWE/PGE toward PAR in BR buffer solution at pH 1.8 with an accumulation time of 35 s was compared to that of PGE by performing the SW-AdSV technique without applying accumulation potential. Relevant SW-AdS voltammograms are shown in Figure 9, which illustrates an obvious PAR peak at 0.1LSWE/PGE, while PGE showed a small response to PAR. The vast difference in responses to PAR between the two electrodes is attributed to the increasing electrochemical area of 0.1LSWE/PGE. Electrochemical areas of PGE and 0.1LSWE/PGE electrodes were calculated using the Randles–Sevcik equation by conducting different measurements at different scan rates and found to be 0.00808 and 0.0136 cm^2^, respectively.

#### 3.4.1 Effect of Scan Rate on LSVs of PAR at 0.1LSWE/PGE

**Figure 10:**
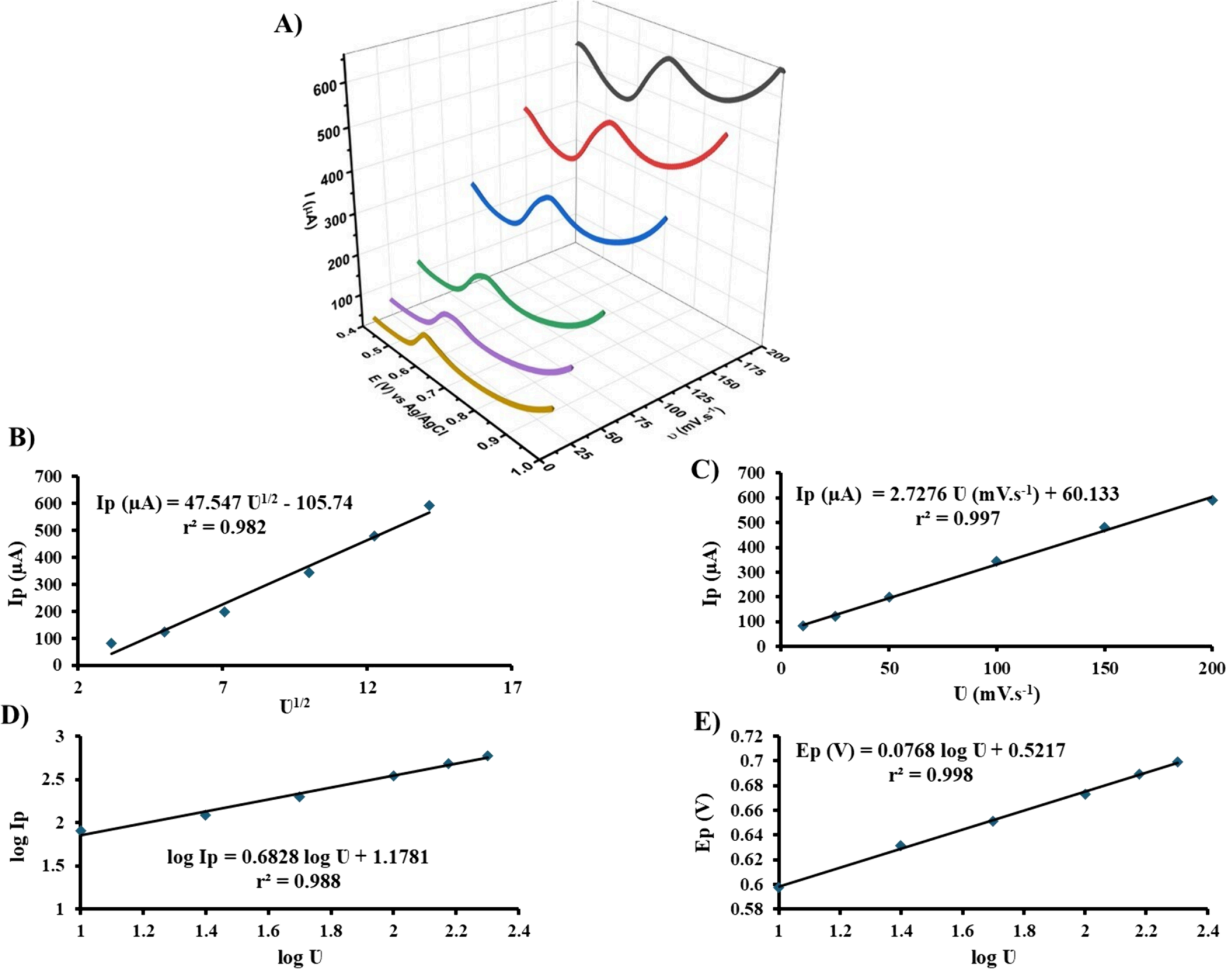
(A) LS voltammograms of 1 mM PAR in BR buffer solution at pH 1.8 and accumulation time 35 s by applying different scan rates 10, 25, 50, 100, 150, and 200 mV s^–1^. (B) Plot of peak current versus square root of the scan rate. (C) Plot of peak current versus scan rate. (D) Plot of log peak current versus log scan rate. (E) Plot of peak potential versus log scan rate.

In order to determine the electrochemical behavior of PAR at 0.1LSWE/PGE, different scan rates of 10, 25, 50, 100, 150, and 200 mV s^–1^ of LSV were applied to 1 mM PAR in a BR buffer solution at pH 1.8 at the potential range from 0.4 to 1.0 V. Relevant linear sweep voltammograms in Figure 10A demonstrated an obvious shifting of PAR oxidation peaks as scan rate increases, which proves the irreversibility of the PAR oxidation reaction at 0.1LSWE/PGE.

The peak currents were observed to increase linearly with both the square root of scan rate and scan rate increases, obeying the linear equations [Ip (μA) = 47.547 υ^1/2^ – 105.74] and [Ip (μA) = 2.7276 υ – 60.133] and linearities *r*^2^ = 0.986 and *r*^2^ = 0.997 as illustrated in Figure 10B,C, respectively. As a result, the PAR electrochemical oxidation kinetics type at 0.1LSWE/PGE was confirmed to be controlled by both diffusion and adsorption processes.

The linear relationship between log (Ip) and log υ was also studied and found to obey the linear equation [log (Ip) = 0.6828 log υ + 1.1781] with linearity *r*^2^= 0.988 and α = 0.68, which is greater than the theoretical value 0.5. This linear relationship confirms that the electrochemical oxidation of PAR at 0.1LSWE/PGE is an adsorption-controlled process. Also, the linear relationship (*r*^2^ = 0.998) between peak potential and log υ with the linear equation [Ep (V) = 0.0768 log υ + 0.5217], confirmed the adsorption process of PAR at 0.1LSWE/PGE.

#### 3.4.2 pH Influence

**Figure 11:**
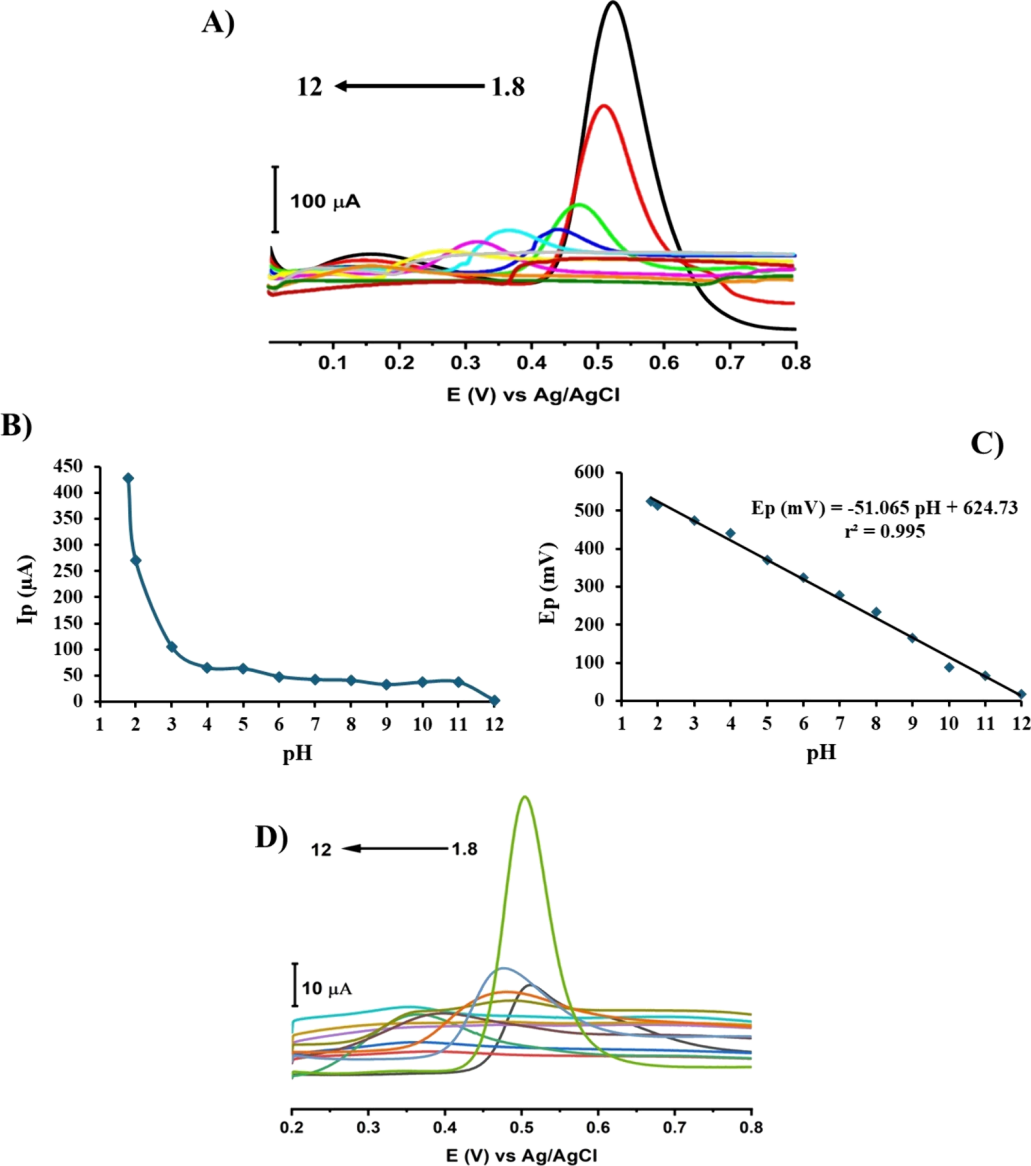
(A) SW-AdS voltammograms of 1 mM PAR in different pHs of BR buffer solution of 1.8, 2.0, 3.0, 4.0, 5.0, 6.0, 7.0, 8.0, 9.0, 10.0, 11.0, and 12.0 and accumulation time of 35 s at 0.1LSWE/PGE. (B) Relevant curve of peak current (μA) versus pH. (C) Relevant curve of peak potential (mV) versus pH. (D) SW-AdS voltammograms of 1 mM PAR in different pHs of BR buffer solution of 1.8, 2.0, 3.0, 4.0, 5.0, 6.0, 7.0, 8.0, 9.0, 10.0, 11.0, and 12.0 and accumulation time of 35 s at PGE.

The pH of the medium was optimized by conducting different measurements for 1 mM PAR in BR buffer solution at different pHs 1.8, 2.0, 3.0, 4.0, 5.0, 6.0, 7.0, 8.0, 9.0, 10.0, 11.0, and 12.0 with an accumulation time of 35 s at 0.1LSWE/PGE by performing the SW-AdSV technique. Figure 11A represents relevant SW-AdS voltammograms. Furthermore, the relationship between peak current (μA) and pH is illustrated in Figure 11B, clarifying that pH 1.8 was accompanied by the highest response to PAR at 0.1LSWE/PGE. Also, as can be seen from Figure 11C, the PAR oxidative peak potential shifts in the negative direction as pH increases, which confirms the involvement of protons throughout the electrochemical oxidation of PAR at 0.1LSWE/PGE. The relationship between peak potential and pH can be expressed as the following equation [Ep (mV) = −51.065 pH + 624.73] with a correlation coefficient of *r*^2^ = 0.995. As oxidation of PAR is associated with the transfer of two electrons and two protons, the slope of the Ep–pH relationship must be 59 mV pH^–1^. The slope obtained in this work 51.065 mV pH^–1^ is close to the theoretical Nernst value with a slight difference; consequently, the electrochemical oxidation of PAR at 0.1LSWE/PGE involves the transfer of the same number of electrons and protons.^55^Figure 11D demonstrates a SW-AdS voltammogram of 1 mM PAR in BR buffer solution at different pHs of 1.8, 2.0, 3.0, 4.0, 5.0, 6.0, 7.0, 8.0, 9.0, 10.0, 11.0, and 12.0 with an accumulation time of 35 s at PGE. Compared to the voltammogram in Figure 11A, the electrochemical response to PAR at 0.1LSWE/PGE is much higher than utilizing PGE without modification, indicating the role of the modification of PGE by 0.1LSWE in enhancing the electrochemical performance toward PAR.

#### 3.4.3 Accumulation Influence

**Figure 12:**
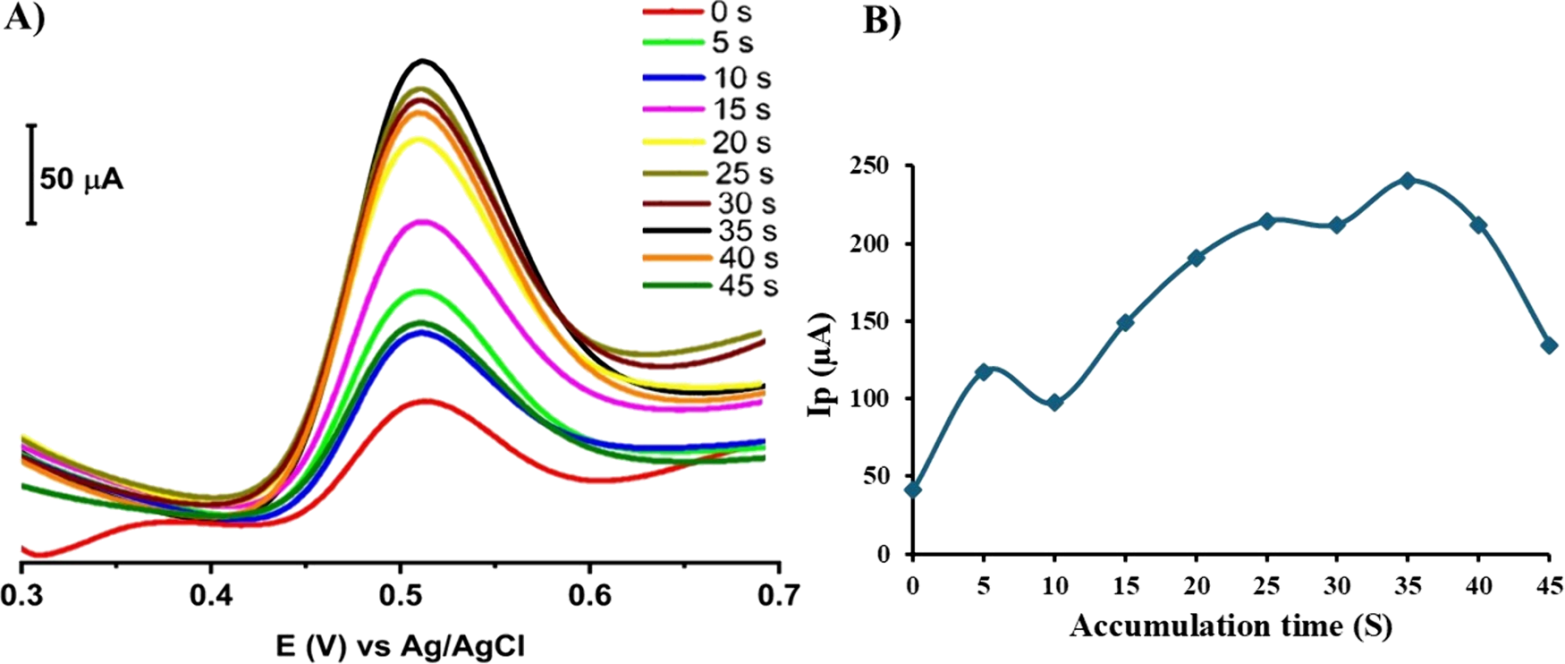
(A) SW-AdS voltammograms of 100 μM PAR in BR buffer solution pH 1.8 at different accumulation times 0, 5, 10, 15, 20, 25, 30, 35, 40, and 45 s at 0.1LSWE/PGE. (B) Relevant plot of peak current (μA) versus accumulation time (s).

SW-AdSV technique involves two key stages. The first stage is the accumulation of PAR at 0.1LSWE/PGE. Subsequently, the accumulated PAR is stripped back into the surrounding solution. Accumulation time is a critical parameter that significantly affects the PAR response intensity at 0.1LSWE/PGE. Hence, optimization of the accumulation time was performed by conducting a series of measurements at varying accumulation times of 0, 5, 10, 15, 20, 25, 30, 35, 40, and 45 s. Figure 12A presents the relevant voltammograms of 100 μM PAR in BR buffer solution at pH 1.8 on 0.1LSWE/PGE, while Figure 12B depicts the plot of peak current (μA) versus accumulation time (s). Applying 35 s of accumulation was accompanied by the highest response for PAR.

#### 3.4.4 Dependence of the Oxidative Peak Current On the PAR Concentration

**Figure 13:**
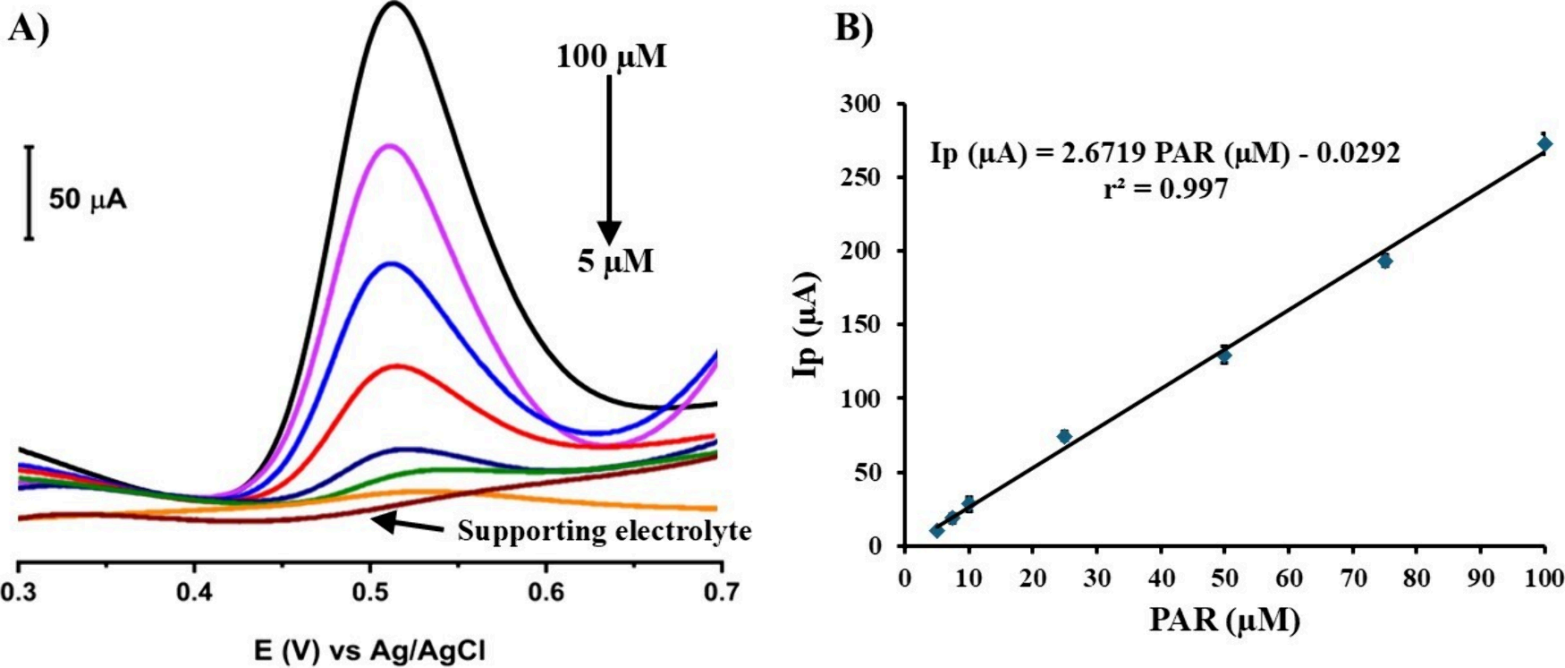
(A) SW-AdS voltammograms of PAR at different concentrations of 5, 7.5, 10, 25, 50, 75, and 100 μM in BR buffer solution at pH 1.8 and 35 s accumulation time at 0.1LSWE/PGE. (B) Relevant standard curve of peak current (μA) versus PAR concentration (μM).

A range of solutions with different PAR concentrations of 5, 7.5, 10, 25, 50, 75, and 100 μM were prepared in BR buffer solution at pH 1.8 and measured by performing the SW-AdSV technique with an accumulation time of 35 s using 0.1LSWE/PGE. A linear relationship was observed between peak current and PAR concentrations within the range 5–100 μM. Figure 13A shows relevant voltammograms, and Figure 13B depicts the corresponding calibration curve of PAR determination with the standard regression equation [Ip (μA) = 2.6719 PAR (μM) – 0.0292] and a high correlation coefficient *r*^2^ = 0.997. Hence, the results confirmed the analytical performance of 0.1LSWE/PGE to determine PAR with a high sensitivity value of 196.46 μA μM^–1^ cm^–2^. The sensitivity was calculated by applying the following equation:
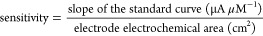


To assess the analytical performance of the proposed method, the limits of detection (LOD) and quantification (LOQ) were determined. These critical values were calculated using the standard deviation of the intercept (σ) and the slope of the standard curve (S) by applying the following equations:
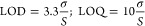


LOD and LOQ were found to be 1.60 × 10^–6^ and 4.51 × 10^–6^ M, respectively. These low values indicate the high sensitivity of the new method for detection of PAR.

Table 2 shows the advantages of the present study in comparison to some relevant literature, highlighting its superior limit of detection (LOD), dynamic range, and sensitivity.

**Table 2:** Comparison between the Present Study and Previous Related Studies Concerning PAR Determination by Voltammetry^a^

voltammetric technique	electrode	LOD (μM)	dynamic range (μM)	sensitivity (μA μM^–1^ cm^–2^)	ref.
DPV	PLPAMCPE	5.4	1.2–12		(39)
DPV	MoO3-GO/CPE	0.39	1–90		(56)
DPV	BiOCl/f-CNT/GCE	0.0016	0.01–650	0.8754	(57)
DPV	Fe–NiO/MCPE	1.84	100–600		(58)
DPV	Au@Fe-MOF/GCE	0.12	0.5–18	4.95	(59)
DPV	Pd/CNTs-Nafion/GCE	0.089	0.2–60	1.532	(60)
DPV	BF/PGE	0.0024	0.05–100		(61)
SWV	PVP/SWCNT/PGE	0.38	1–500		(62)
SW-AdSV	0.1LSWE/PGE	1.6	5–100	196.46	this work

a PLPAMCPE: l-phenylalanine modified carbon paste electrode, MoO3-GO: MoO_3_ nanobelt-graphene oxide, f-CNT: functionalized carbon nanotube, Fe–NiO: ferric doped nickel oxide, Pd/CNTs: carbon nanotubes supported Pd nanoparticles, BF: 5,5×-(oxybis(4,1-phenylene))bis(3-(2-hydroxyphenyl)-1-phenylformazan, SWCNTs: single-wall carbon nanotubes, PVP: polyvinylpyrrolidone.

### 3.5 Interference Influence

The anti-interference ability of the proposed methodology was studied in the presence of a series of organic and inorganic chemicals. A 100-fold excess of dopamine, uric acid, caffeine, and ascorbic acid was spiked into a 50 μM PAR in BR buffer solution at pH 1.8 and measured by the SW-AdSV technique and applying an accumulation process of 35 s. Similarly, the impact of different inorganic salts (Na^+^, K^+^, Mg^2+^, Ca^2+^, NO_3_^–^, and Cl^–^) at 100-fold excess on the 50 μM PAR peak current was investigated. No significant influence was observed on the PAR peak current for any of the investigated interferants, which indicates the capability of the methodology to function effectively in matrices containing those chemicals without compromising its performance.

### 3.6 Application of 0.1LSWE/PGE in the Determination of PAR in Real Pharmaceutical Samples

**Figure 14:**
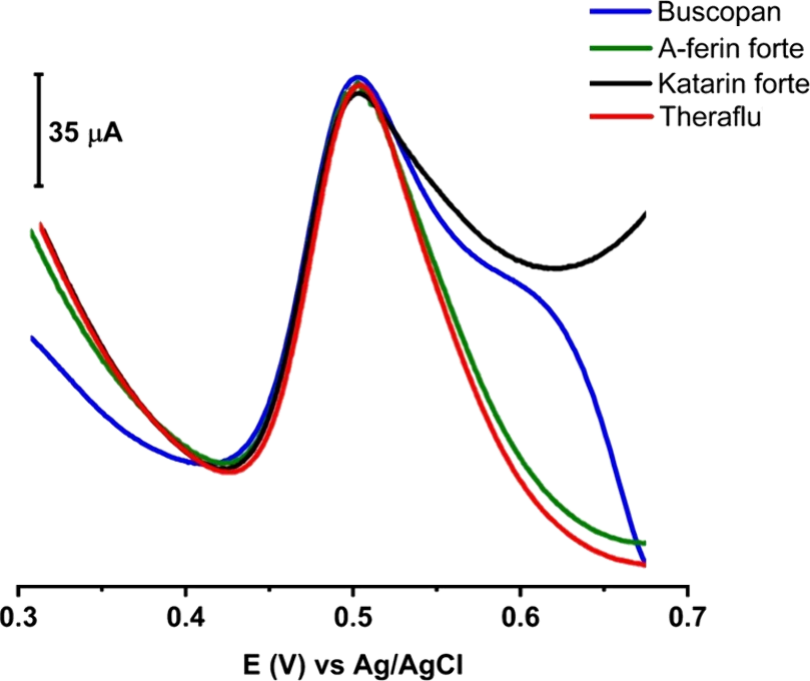
SW-AdS voltammograms of PAR containing pharmaceuticals prepared in BR buffer solutions at pH 1.8 by applying 0.1LSWE/PGE and an accumulation time of 35 s and a scan rate of 100 mV s^–1^.

To evaluate the ability of the novel electrode and methodology to apply in real samples, different PAR-containing pharmaceutical brands (Buscopan, A-ferin forte, Katarin forte, and Theraflu) were prepared and measured by employing SW-AdSV at 0.1LSWE/PGE as demonstrated in Figure 14. Table 3 demonstrates the prepared and found concentrations of PAR in the tested pharmaceutical samples. The recovery was within the range 99.76 −102.87%, which validated the applicability of the methodology to determine PAR in different pharmaceutical matrices.

**Table 3:** Prepared and Found Concentrations of PAR in Real Pharmaceutical Samples and Their Recovery % by Applying 0.1LSWE/PGE

pharmaceutical trade name	prepared concentration (μM)	found concentration (μM)	recovery %
Buscopan	50	51.32	102.63
A-ferin forte	50	50.54	101.08
Katarin forte	50	49.88	99.76
Theraflu	50	51.44	102.87

## 4 Conclusions

This work introduces a facile, sensitive, selective, and low-cost methodology that was investigated to determine PAR in various pharmaceutical samples. The new methodology utilizes the SW-AdSV technique at PGE modified by LSWE. Furthermore, this study considers the first application of LSW in electroanalytical chemistry. The extraction process of LSW was optimized, 0.1 g of LSW in 10 mL of acetonitrile as an extraction ratio yields the best electrochemical response toward PAR upon modification of the PGE. Moreover, optimization of the method involved evaluating the influence of pH and accumulation time on the PAR response. Employing the BR buffer solution at pH 1.8 and an accumulation time of 35 s resulted in the highest PAR peak current. A linear relationship between PAR peak current and PAR concentration was found to be within the range of 5–100 μM with a correlation coefficient (*r*^2^) of 0.997. LOD and LOQ were determined to be 1.60 and 4.51 μM, respectively. The proposed methodology was successfully applied to determine PAR in real pharmaceuticals.

## Notes

This research is considered as a part of the PhD thesis of Wael Bosnali.
